# A Tomato EMS-Mutagenized Population Provides New Valuable Resources for Gene Discovery and Breeding of Developmental Traits

**DOI:** 10.3390/plants11192453

**Published:** 2022-09-20

**Authors:** Rocío Fonseca, Carmen Capel, Roberto Nieto-Canseco, Ana Ortiz-Atienza, Sandra Bretones, Juan D. López-Fábregas, Abraham S. Quevedo-Colmena, Ricardo Lebrón, Teresa Barragán-Lozano, Víctor Villalobos-Ramírez, Fernando J. Yuste-Lisbona, Trinidad Angosto, Juan Capel, Rafael Lozano

**Affiliations:** Centro de Investigación en Biotecnología Agroalimentaria (BITAL), Universidad de Almería, 04120 Almería, Spain

**Keywords:** tomato, chemical mutagenesis, gene functional cloning, *SlARF10A*, breeding

## Abstract

Tomato (*Solanum lycopersicum* L.) is a major horticultural crop and a model species among eudicots, especially for traits related to reproductive development. Although considerable progress has been made since the tomato genome sequence project was completed, most of the genes identified remain predictions with an unknown or hypothetical function. This lack of functional characterization hampers the use of the huge amount of genomic information available to improve the quality and productivity of this crop. Reverse genetics strategies such as artificial mutagenesis and next-generation sequencing approaches build the perfect tandem for increasing knowledge on functional annotation of tomato genes. This work reports the phenotypic characterization of a tomato mutant collection generated from an EMS chemical mutagenesis program aimed to identify interesting agronomic mutants and novel gene functions. Tomato mutants were grouped into fourteen phenotypic classes, including vegetative and reproductive development traits, and the inheritance pattern of the identified mutations was studied. In addition, causal mutation of a selected mutant line was isolated through a mapping-by-sequencing approach as a proof of concept of this strategy’s successful implementation. Results support tomato mutagenesis as an essential tool for functional genomics in this fleshy-fruited model species and a highly valuable resource for future breeding programs of this crop species aimed at the development of more productive and resilient new varieties under challenging climatic and production scenarios.

## 1. Introduction

Global agriculture systems and food production currently face significant issues. Mainly, a global population likely to grow to nearly 10 billion people by 2050 makes it necessary to achieve increased crop productivity capable of facing this demographic growth with minimal environmental impact. The development of new crop varieties with desirable agronomical traits, such as high productivity rates, has become crucial for this purpose. Since natural variation is scarce, breeding programs are in need of suitable alternatives and particularly induced mutagenesis programs [1]. Modern breeding programs should ideally be based on a combination of natural and induced mutagenesis supported by advanced molecular biology techniques [2].

Tomato (*Solanum lycopersicum* L.) is one of the major crop species whose global demand has increased over the last decades [3], mostly due to its diverse forms of consumption (fresh or processed) and its highly nutritive values, including mineral content [4]. Tomato is considered to be beneficial to health since it is a rich source of antioxidants and beta-carotene, as well as vitamins [5,6], and has been linked to cardiovascular disease prevention [7]. This species is a good example of the successful use of spontaneous mutations in breeding programs. Indeed, mutations such as *self-pruning* (*sp*) [8], *ovate* (*o*) [9], or *jointless* (*j*) [10] are widely used in the processing industry to obtain determinate compact growing plants, with elongated fruits and more suitable for mechanical harvesting. Furthermore, other mutations such as *fasciated* (*fas*) [11] and *locule number* (*lc*) [12], which control a trait of great agronomic importance, such as fruit size, are essential tools in breeding programs of fresh market varieties. In addition to its economic importance, tomato is considered a model species among fleshy fruit plants due to its favorable agronomic traits, such as a short life cycle or self-pollination, as well as biotic and abiotic stress resistance [13,14,15]. Likewise, the tomato genome is relatively small (~900 Mb), and its full sequence is available, which facilitates the genomic breeding of this species [16]. Nevertheless, only a small percentage of the nearly 34,075 genes annotated in the tomato genome has an associated function [17]. The Tomato Genetics Resource Center at the University of California, Davis (http://tgrc.ucdavis.edu/ accessed on 7 July 2022), regarded as the most important seed stock center of tomato, contains phenotypic information on 1050 monogenic mutants at 630 putative genetic loci. Since natural variation does not seem to provide enough resources for tomato’s functional genomics, mutational analysis is revealed as a suitable strategy for gene functional characterization and breeding programs. 

Induced mutagenesis techniques available include insertional mutagenesis as well as the use of physical and chemical agents that have very diverse effects on target genomes. Insertional mutagenesis methods such as T-DNA have been successfully employed in tomato using methods such as activation tagging [18,19], but the need for transformation using *Agrobacterium tumefaciens* remains a time-consuming task. Commercial backgrounds such as the Moneymaker cultivar (MM) have been used for the generation of an enhancer trap T-DNA collection by *Agrobacterium*-mediated transformation [20]. Although these mutant collections represent a highly valuable resource for functional analyses in this model species, their potential use as breeding lines is limited since they are transgenic plants. Among the physical agents, fast neutrons have been used in *Arabidopsis thaliana* and rice (*Oryza sativa*), where a total of 51,840 and 10,000 mutant lines, respectively, have been described [21,22]. Nevertheless, fast neutrons cause huge deletions or even the complete loss of entire chromosomes, which diminishes mutant seeds’ viability [23]. Other physical agents include gamma- and X-rays as well as UV light, whose effects range from small modifications to large deletions [24,25,26]. 

Alkylating agents such as ethyl methanesulfonate (EMS) are highly effective and relatively easy to handle. EMS commonly alkylates guanine (G), rendering O6-etylguanine, which pairs with thymine (T) instead of cytosine (C), and as a result, C/G to T/A transitions occur. Nevertheless, G/C to C/G or G/C to T/A transversions by 7-methylguanine hydrolysis are also produced, although with a lower frequency [27]. This chemical mutagen has been used to generate tomato mutant collections, being the largest one that obtained in the cultivar Micro-Tom [28]. This cultivar was developed for garden purposes, and it is characterized by a dwarf phenotype determined by a combination of mutations affecting hormone perception as well as a determinate growth habit [29]. The small size of Micro-Tom makes it suitable for the cultivation of a large number of plants in a reduced space, and a relatively short life cycle allows to obtain M_2_ seeds faster. Watanabe et al. (2007) [30] have carried out a characterization of 3839 mutant M_2_ lines obtained by EMS mutagenesis in the Micro-Tom genetic background, which allowed them to identify a broad range of mutants. Furthermore, 8598 Micro-Tom mutant lines have been characterized and added to a database called TOMATOMA (https://tomatoma.nbrp.jp/ accessed on 7 July 2022), which integrates mutant phenotypes as well as associated data, making this information available for consult [31]. Nevertheless, cultivars different from Micro-Tom may be more appropriate for the identification of genes related to plant size or vigor as well as fruit size [20]. Other genetic backgrounds have been used such as the M82 cultivar, with the advantage that it can be grown as a determinate plant in an open field since it carries the *sp* mutation. This cultivar has been employed by Menda et al. (2004) [32] in the generation of 3417 EMS mutant lines, which provided new isogenic alleles of monogenic mutants already cataloged in the Tomato Genetics Resource Center (https://tgrc.ucdavis.edu/ accessed on 7 July 2022), although it also included new ones. 

EMS mutagenesis combined with high-throughput methods for detecting point mutations such as TILLING are powerful tools for reverse genetics in tomato [33], but they are not suitable for the identification of genes that control phenotypes never before described. With the aim to characterize new gene functions and mutant alleles in loci of agronomical interest, we have carried out a mutagenesis program using EMS in seeds of the tomato MM cultivar, which has allowed us to obtain a mutant collection that comprises more than 8000 mutant lines. This mutant collection has been screened in this work under greenhouse conditions, and a large number of new mutant alleles have been identified. As a proof of concept, the mapping-by-sequencing approach was used to identify the causal mutation of a selected mutant, proving that EMS-induced mutagenesis combined with this mapping strategy is a robust approach for the identification of key regulators in plant growth and reproductive development.

## 2. Results

### 2.1. Development of EMS Mutant Collection

**Figure 1:**
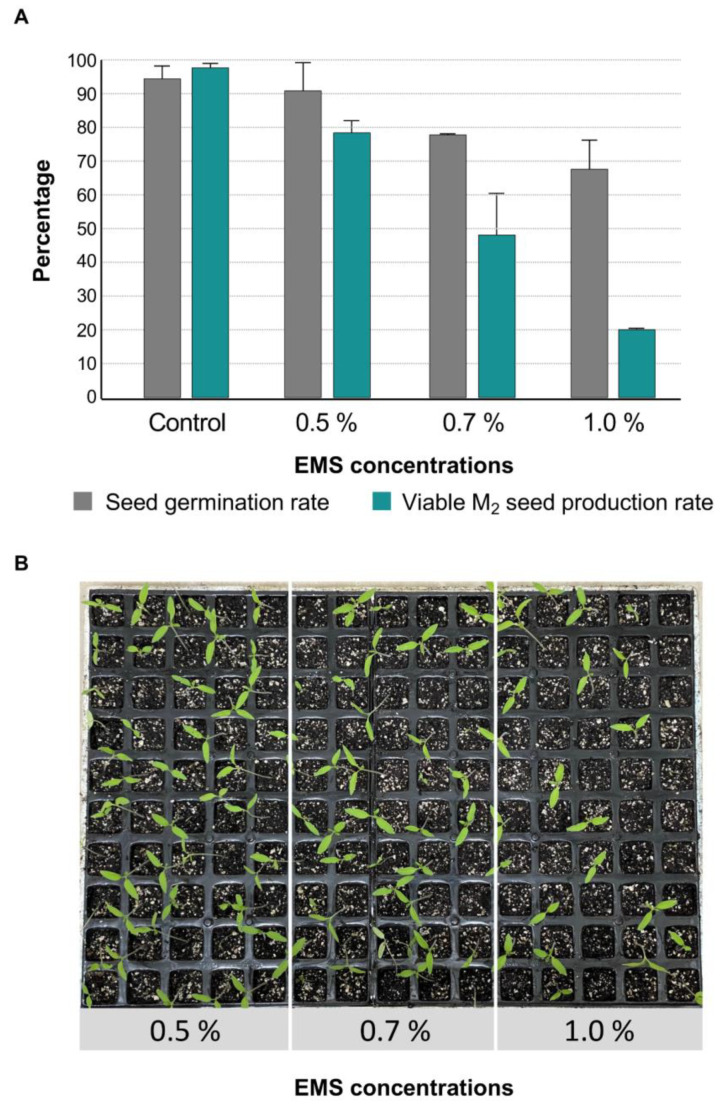
Calculation of the mutagen median lethal dose (LD_50_) in three treatments of 0.5%, 0.7%, and 1.0% of EMS concentration. (**A**) Percentage of seed germination rate and M_2_ offsprings obtained from M_1_ plants that germinate are shown for each treatment. (**B**) Seedlings developed from each of the three EMS concentrations assessed.

EMS treatment was performed from tomato seeds of the Moneymaker cultivar (MM), a commercial background that exhibits indeterminate growth and yields medium size fruits. To optimize the mutagenesis protocol, the mutagen median lethal dose (LD_50_) was established as a preliminary step, given that it would permit to obtain of an appropriate number of mutant lines without compromising seed viability and sterility. With this aim, three treatments using different EMS concentrations, i.e., 0.5%, 0.7%, and 1.0%, were carried out with a total number of 500 seeds for each treatment. The results obtained in these experiments are shown in Figure 1.

**Figure 2:**
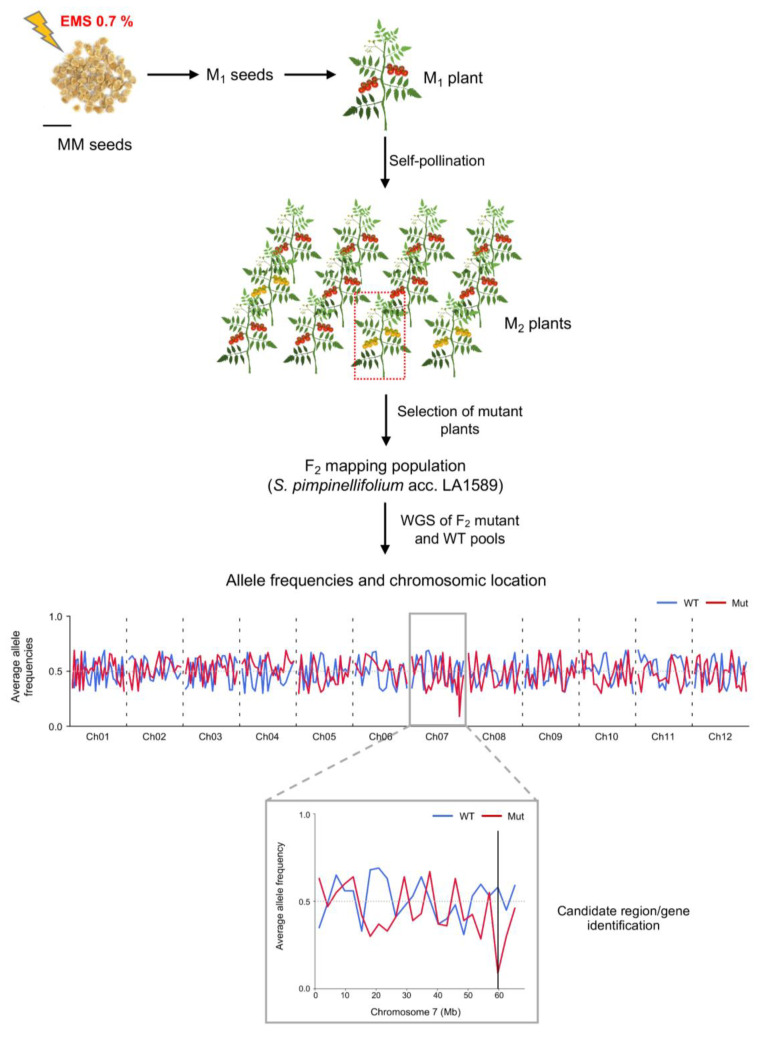
Overview of the mutagenesis procedure followed to identify tomato mutants and to isolate the corresponding causal genes. First, Moneymaker (MM) seeds were exposed to 0.7% of EMS concentration for 16 hours, and after sowing, M_1_ plants were phenotypically characterized in order to detect dominant mutations and then self-pollinated to obtain M_2_ progenies from which recessive mutants were identified. In addition, an F_2_ interspecific population derived from the cross of a given tomato mutant (MM background) with *Solanum pimpinellifolium* accession LA1589 as pollen donor was obtained. Subsequently, whole-genome sequencing (WGS) of F_2_ wild-type (WT) and mutant (Mut) DNA pools was performed for mapping-by-sequencing experiments aimed at identifying the candidate chromosome region and the gene responsible for a given mutant phenotype (for details, see Yuste-Lisbona et al., 2021 [34]). Scale bar applies for 1 cm.

As the EMS concentration increased, the germination of M_1_ seedlings decreased (Figure 1B), and therefore, the delay in seed germination and seedling development was related to the EMS level. For each treatment, the number of M_1_ seeds that germinate and produce viable M_2_ progenies was also assessed. At 0.5% of EMS concentration, the greatest germination and fertility rates (percentage of plants that produce viable M_2_ seeds), 91.95% and 79.20%, respectively, were observed. The opposite results were obtained using 1.0% of EMS concentration, as only 20.70% of the mutagenized seeds produced M_2_ progenies. The seed germination and fertility rates reached, respectively, 78.5% and 48.8% at 0.7% of EMS concentration, which was considered the lethal dose LD_50_ in our experimental conditions (Figure 1). Therefore, a large-scale mutagenesis experiment was performed at 0.7% of EMS concentration with MM seeds to produce M_2_ progenies, as shown in Figure 2. 

### 2.2. Phenotypic Screening of EMS Mutant Lines

A total number of 16,104 MM seeds were EMS treated during the development of the mutant collection, and 12,560 M_1_ plants were obtained, from which 7379 (58.75%) produced M_2_ segregating progenies while the remaining ones were unable to develop fertile flowers and hence, did not yield M_2_ offspring. Thus, a high proportion of M_1_ plants (5181 plants, 41.25%) was found to show developmental alterations affecting either vegetative or reproductive traits, suggesting that they accumulated deleterious mutations. All the M_2_ progenies obtained were screened under greenhouse conditions along eight crop cycles between spring–summer 2014 and autumn–winter 2018 in order to detect recessive mutants as well as corroborate putative dominant mutants from which seeds were obtained. With this aim, 10–16 seedlings of each M_2_ family were transplanted and cultivated; thus, 97,104 M_2_ plants were screened for alterations in visible phenotypes. A total of 2800 mutants (37.95%) were detected and grouped into fourteen phenotypic categories depicted in Table 1. These categories included vegetative traits such as seedling and root development, plant growth and branching, leaf color, morphology, and senescence of the leaves, as well as reproductive traits such as flowering time, inflorescence architecture, flower development, fruit development and ripening, parthenocarpy, and fruit cuticle development. 

**Figure 3:**
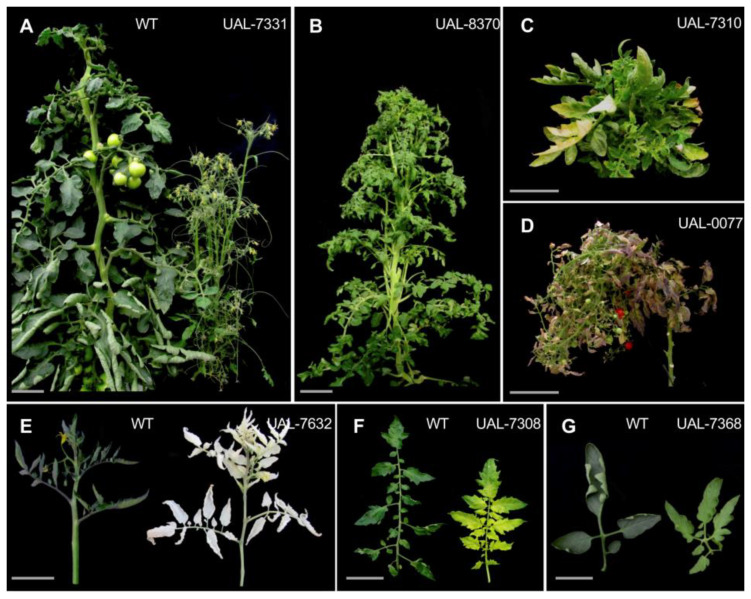
EMS mutant lines displaying alterations during vegetative development and belonging to different phenotypic classes (see text and Table 1 for further details). Scale bars: 2 cm in (**A**,**B**,**F**,**G**), and 5 cm from (**C**–**E**).

Some of the mutants that exhibited vegetative development alterations are shown in Figure 3. As an example, mutants altered in plant architecture (UAL-7331 line, Figure 3A) and branching (UAL-8370, Figure 3B), both included in phenotypic class III, as well as others showing dwarfism and compact growth (UAL-7310 and UAL-0077 lines, class III, Figure 3C,D, respectively) were identified. Additionally, extreme albinism affecting vegetative organs was detected in the mutant line UAL-7632 (Figure 3E), whereas chlorosis and leaf color phenotypes were detected in lines UAL-7308 (Figure 3F) and UAL-7368 (Figure 3G), respectively, all of them included in phenotypic class IV (Table 1). 

**Figure 4:**
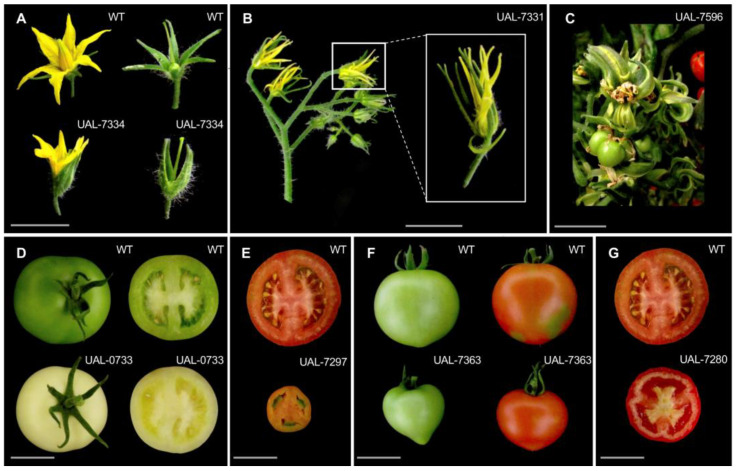
EMS mutant lines showing alterations in reproductive development and belonging to different phenotypic classes (see text and Table 1 for further details). Scale bars: 1 cm from (**A**–**C**) and 5 cm from (**D**–**G**).

Regarding reproductive development, parthenocarpy (class XII) and fruit morphology and color (class XI) account for the highest number of mutants (8.54% and 8.18%, respectively). Some of these mutant lines are shown in Figure 4, including UAL-7334 (Figure 4A), which displayed abnormally fused sepals; UAL-7331, characterized by a typical *wiry*-like phenotype including tiny floral organs (Figure 4B), and UAL-7596 (Figure 4C) showing severe homeotic flower alterations. All three belong to phenotypic class VIII (Table 1). Mutant lines altered in fruit color (UAL-0733, Figure 4D), as well as those showing fruit shape and size abnormalities (Figure 4E–G), are also shown as representatives of phenotypic class XI. 

Reproducibility of phenotyping was further assessed on M_3_ progenies as a verification step, particularly when only one mutant plant was identified in a given M_2_ family. Thus, during the characterization of the EMS population, a total of 217 M_3_ families were screened. Out of them, 187 M_3_ families showed the same phenotype previously found in the M_2_, while 19 mutant lines did not segregate for the selected phenotype, and 11 M_3_ families showed new phenotypes, suggesting that a huge number of mutations are segregating in these families and that some of the new phenotypes may be due to gene interactions, including epistasis.

On the other hand, some mutant phenotypes resembled other ones also identified in our EMS mutant collection. To determine putative mutant alleles of a given gene, complementation tests were performed in 137 mutant lines showing phenotype similarities to any of the phenotypic categories included in Table 1, in particular those related to plant architecture and branching, flower morphology, inflorescence architecture, and fruit ripening. As a result, 19 mutant lines were included in 8 complementation groups. Some of these mutant lines found to carry allelic mutations are UAL-7331 and UAL-7972, both characterized by a *wiry*-like phenotype which is shown in Supplementary Figure S1A–C. 

In addition, a genetic complementation analysis was also carried out for some specific lines suspected to be allelic of other mutants previously reported in the scientific literature. This is the case of the UAL-0733 mutant line, whose fruits are pale-yellow at the mature green stage (Supplementary Figure S1D–I). The *lutescent* mutants of tomato, *lutescent1* (*l1*) and *l2*, are nonallelic monogenic mutants that show a progressive loss of chlorophyll from leaves and fruits [35,36,37]. In *l1* and *l2* fruits, the early loss of chlorophyll during development yields fruits of a whitish-yellow color prior to the onset of ripening [35]. Complementation tests performed by crossing UAL-0733 with the *l1* or *l2* mutants proved that the mutation detected in line UAL-0733 was allelic to *l1*.

### 2.3. Gene Isolation of Causal Mutations by Whole Genome Sequencing Approach

Regarding newly identified phenotypes, functional genomics studies require an efficient method to identify the mutated gene in any selected line. With this aim, we implemented an efficient method based on a mapping-by-sequencing strategy through the development of F_2_ mapping populations derived from the cross of mutant lines and *S. pimpinellifolium* accession LA1589 as pollen donor. Thus, a mapping-by-sequencing strategy was carried out by sequencing DNA pools formed by equimolar amounts of DNA from mutant and wild-type (WT) F_2_ plants. Analysis of allele distributions in both genomic DNA pools allows for the physical mapping of the causal mutation in the genome position where the allele frequency of the mutant pool reaches zero [34]. Finally, the identified candidate genomic region is filtered for unique mutations in homozygous state, among which will be the causal mutation responsible for the selected phenotype. An overview of the whole process, including mutagenesis and identification of causal mutations, as well as allele frequency detection, is shown in Figure 2.

**Figure 5:**
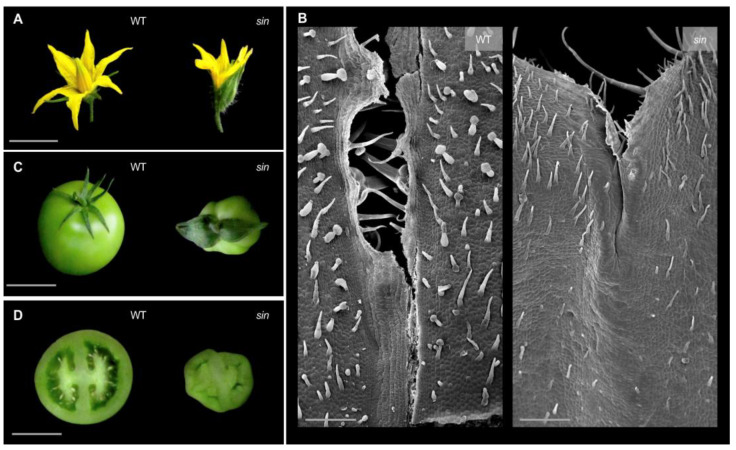
Phenotype of the *sepal indehiscent* (*sin*) mutant line (UAL-7334). (**A**) Wild-type (WT) pentameric Moneymaker flowers and mutant flowers showing fused sepals. (**B**) Scanning electron microscopy (SEM) performed on sepals of WT and *sin* mutant flowers at pre-anthesis displaying morphological differences in the epidermal cells located in the abscission zone between each sepal. (**C**) Mutant plants also developed smaller and parthenocarpic (seedless) fruits when compared to WT ones (**D**). Scale bars: 0.5 cm in (**A**,**C**,**D**) and 250 µm for (**B**).

The mutation responsible for the mutant phenotype observed in the UAL-7334 line (Figure 5) is among those selected to identify the causal gene by the described mapping-by-sequencing approach, which was used as proof of concept of this strategy’s successful implementation. UAL-7334 mutant plants exhibited a fusion in sepals that develop in a bilateral axis and not with the radial symmetry characteristic of tomato flowers (Figure 5A). A closer view of sepals using scanning electron microscopy (SEM) proved that sepals of mutant plants lack the abscission zone between each sepal, which is typical of WT plants, and as a result, they remain fused until mechanical tensions derived from fruit growth separate them (Figure 5B). In addition, mutant plants develop smaller fruits that are parthenocarpic or have a lower number of seeds when compared to WT plants (Figure 5C,D). The segregation ratio observed in the M_2_ progeny of the line UAL-7334 was consistent with a monogenic recessive inheritance for the mutant phenotype (87 WT: 33 mut; χ^2^ = 0.4, *p* = 0.52). Given the phenotype observed in the sepals of mutant plants, we have called this mutant *sepal indehiscent* (*sin*). 

**Figure 6:**
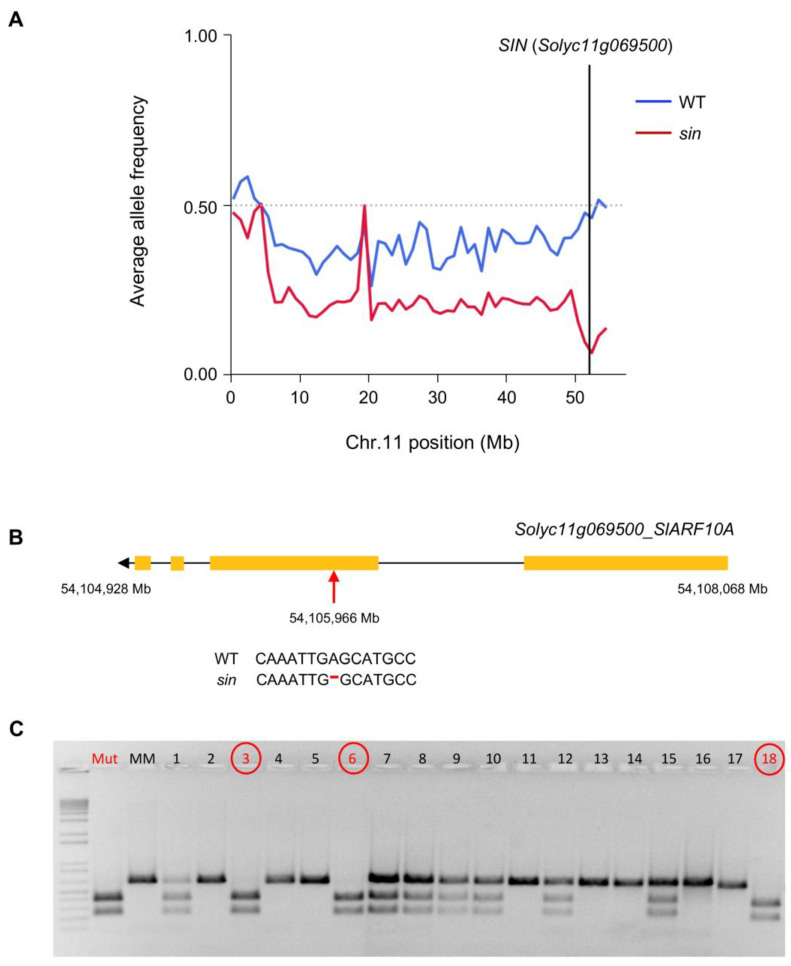
Whole-genome sequencing performed on wild-type (WT) and *sepal indehiscent* (*sin*) mutant F_2_ DNA pools from an interspecific F_2_ mapping population of the *sin* mutant. Allele frequency analysis and chromosome location of the *sin* mutation (**A**), consisting of a frameshift deletion in the second exon of *Solyc11g069500*, an auxin response factor (*SlARF10*) (**B**). (**C**) Co-segregation test performed in an M_2_ segregating family confirmed this mutation in *Solyc11g069500* as responsible for the *sin* mutant phenotype. Red circled numbers indicate plants displaying mutant phenotype.

With the aim of detecting the chromosomic location of the *sin* mutation, an F_2_ mapping population was obtained derived from the cross of a *sin* mutant plant with pollen of *S. pimpinellifolium* (LA1589 accession). Sequencing of the pools, each composed of DNA from 15 F_2_ mutant and 15 F_2_ WT plants, allowed us to determine that causal mutation was located in chromosome 11 (Figure 6A). Variant analysis of a 5 Mb interval encompassing the candidate region located at the end of chromosome 11 led to the identification of 13 homozygous polymorphisms in the mutant pool, which had not been previously reported in the sequenced tomato genomes. The assessment of the functional impact of these candidate causal mutations showed that only one is located in an exonic region promoting functional effects in protein-coding sequence. Particularly, a single nucleotide deletion in the coding region of the gene *Solyc11g069500* that codes for an Auxin Response Factor (SlARF10A) was detected in this candidate region (Figure 6B). Such deletion causes a frameshift giving rise to an aberrant protein that keeps the proximal DNA B3 binding domain and the medium auxin response factor functional domain, although it lacks the carboxy terminal dimerization domains (DTD) Aux/IAA, involved in protein-to-protein interactions and required for the binding to Aux/IAA proteins [38,39] (Supplementary Figure S2). Interestingly, Damodharan et al. (2018) [40] have described that CRISPR-Cas9 knock-out lines of this gene showed alterations in leaf and flower development which included the fusion of sepals. With the aim to support the causal relationship between the mutation identified and the *sin* mutant phenotype described above, a co-segregation analysis of the detected mutation with the mutant phenotype was assessed in the M_2_ segregating population previously phenotyped. All 33 *sin* mutant plants were homozygous for the *Solyc11g069500* mutation, whereas 87 WT plants were heterozygous (61 plants) or homozygous (26 plants) for the WT allele (χ^2^ = 0.85, *p* = 0.65). A co-segregation test and the fact that EMS mutant line phenocopies knock-out lines previously described [40] support the hypothesis that the mutant phenotype was caused by the identified mutation in the *SlARF10A* gene (Figure 6C). Identification of the causal gene of the mutation detected in this EMS line is proof of concept of this methodology’s utility for the genomic characterization of EMS mutant lines.

## 3. Discussion

Since the first version of the tomato genome sequence was available [16], huge progress has been made in the understanding of genome organization. In that version of the tomato genome, the International Tomato Annotation Group (ITAG) predicted 34,727 protein-coding gene models, but in a few years, hundreds of tomato accessions have been re-sequenced [41,42], and large amounts of transcriptomic data related to key developmental processes such as fruit ripening or plant development under abiotic stress conditions have been made available [43,44]. The most recent version of the tomato genome includes 34,075 annotated-protein-coding genes [17], from which only 29,532 have a functional description based on expression evidence support or on homology with plant protein databases which, in turn, are also predicted proteins. In conclusion, most of the tomato genes have been predicted by in silico analysis, yet their functions remain unknown or hypothetical. Tomato mutants have been and are still directly used in breeding programs [8,10,45], but natural variation of agronomically interesting alleles is scarce in natural germplasm. Thus, induced mutagenesis rises as one of the most relevant genomics tools to allow for the functional characterization of most of the unknown or uncharacterized tomato genes. Moreover, induced mutagenesis allows to create new allelic variants in a short period of time with the aim to shed light on gene functions through a reverse genetics’ strategy [46] or to incorporate new germplasm into breeding programs. Indeed, several tomato mutant collections have been generated over the last few years using both chemical [31], physical [21,22], and biological [20] mutagens. 

Chemical mutagenesis and particularly alkylating agents such as EMS have been widely employed in crop species. In rice (*O. sativa*), this strategy has allowed obtaining mutant lines displaying different phenotypes related to agronomical traits of interest such as the photosynthetic rate of leaves [47], abiotic stress tolerance, or drought resistance based on root length and volume [48] as well as a heat tolerant mutant that exhibits higher photosystem II efficiency [49]. In maize (*Zea mays*), mutations induced by EMS affecting embryo morphogenesis have been described, whose main effects range from reduced germination, delayed development, and aberrant morphology to lethality [50]. Furthermore, the identification of two maize EMS-induced mutations related to dwarf, and pale-green phenotypes, respectively, has been carried out by Heuermann et al. (2019) [51]. All these studies demonstrate the potential use of EMS mutagenesis for higher yield and stress resistance in grasses. 

In this study, the tomato MM commercial cultivar has been used for EMS mutagenesis. Plants of this genetic background show an indeterminate growth and a 4–6-month life cycle, such as most of the fresh market commercial varieties. Given that, time and considerable greenhouse space have been consumed in the characterization of this mutant collection. Some of the most relevant vegetative and reproductive phenotypes detected are depicted in Figure 3 and Figure 4 and account mainly for vegetative development traits related to overall plant growth (42.71%) and seedling lethality and albinism (9.04%), as well as for reproductive development traits such as parthenocarpy (8.54%) and fruit morphology and color (8.18%). Thus, the EMS mutant collection hereby characterized offers a valuable resource for tomato breeding, not only because it is the first collection to be developed in a fresh-market variety, unlike previously described EMS collections that have been developed in the small determinate growing cultivar Micro-tom [31], but also due to the large number of mutant lines characterized and the fact that mutant alleles can be directly included in breeding programs since these are non-transgenic plants developed in a commercial background.

Furthermore, the identification of novel mutations performed by mapping-by-sequencing has led to the isolation of causal mutations of agronomically interesting mutant lines. Such is the case of the line UAL-7334 (*sin*), where this approach has allowed us to relate a single nucleotide deletion in the *Auxin Response Factor 10A* (*SlARF10A*) gene to this mutant phenotype. This gene has been reported as belonging to a family involved in the auxin perception pathway [52]. According to their effect on the expression of auxin-responsive genes, ARFs can be considered activators or repressors based on the amino acid composition of the ARF’s middle region domain [53]. SlARF10A has been reported to act as a transcriptional repressor in a similar way to its *Arabidopsis* and rice homologs [53] and has been related to a broad spectrum of biological processes, such as control of leaf shape and fruit chlorophyll content [54] or fruit sugar accumulation [55]. Moreover, a key role in leaf lamina outgrowth and floral organ development has been described for this gene through a CRISPR-Cas9 knock-out strategy [40]. These CRISPR lines developed compound leaves that lack the serrations common to WT; also, the leaf area and leaflets blades were bigger. Flowers also displayed severe abnormalities, and sepals of these knock-out lines appear fused in a similar way to the phenotype observed for the *sin* mutant as a result of lamina outgrowth [40]. The wide range of phenotypes observed by these authors may be related to the fact that these knock-out lines bore severe alleles, which resulted in frame shifts leading to premature protein termination after 35–50 amino acid residues; thus, these peptides lack all functional domains of SlARF10A. On the other hand, the EMS-induced mutation detected in the *sin* mutant could be considered a less severe or leaky allele since protein termination occurs at the 442 amino acid residues, producing a protein that only lacks the carboxy terminal Aux/IAA dimerization domain. Taken together, all these results conclude that *SlARF10* is essential to inhibit lamina outgrowth as well as to normal patterning of leaves and flowers.

Apart from the *sin* mutant, the causal mutations of other striking phenotypes have been identified by using a mapping-by-sequencing approach in which the wild tomato species *S. pimpinellifolium* is outcrossed with the mutant of interest to generate an F_2_ interspecific mapping population. The use of another species genetically distant from cultivated tomato provides a huge number of polymorphisms for linkage analysis, simplifying the bioinformatic workflow, and ensures a high mapping accuracy, thus facilitating the discovery of novel genes of agronomic interest. Such is the case of *succulent stamens 2* (*sus2*), which exhibits a stamen aberrant morphology as a result of a complete homeotic change into carpels that turns in a total absence of pollen and the development of parthenocarpic fruits [56]. Thus, this tomato male sterile mutant is a very useful tool for programs of hybrid seed production. Also remarkable is the *hairplus* (*hap*) mutant, which shows an increase in trichome density in stems and inflorescence stems [57], an extremely interesting agronomic trait widely related to biotic stress resistance such as plagues [58]. However, the use of wild tomato species for a mapping-by-sequencing forward genetics approach may mask some phenotypic traits, such as small-fruited mutations. In these instances, a mapping population created by backcrossing a mutant plant to its non-mutagenized parent can be used, as described by Garcia et al. (2016) [59]. Taken together, all our findings provide new evidence on the key role of mutagenesis and mutant analysis as a valuable tool for tomato functional genomics. Furthermore, given that our mutant collection has been obtained through a chemical mutagenesis approach, all the identified mutations reveal themselves as suitable for tomato breeding programs.

## 4. Material and Methods

### 4.1. Plant Material and Growth Conditions

Seeds of tomato (*S. lycopersicum*, Moneymaker cultivar, accession LA2706) and the wild relative species *S. pimpinellifolium* (accession LA1589) were obtained from Tomato Genetics Resource Center (TGRC, http://tgrc.ucdavis.edu/ accessed on 7 July 2022). Moneymaker (https://tgrc.ucdavis.edu/Data/Acc/AccDetail.aspx?AccessionNum=LA2706&contains=false accessed on 1 August 2022) is an indeterminate growing cultivar that produces medium size, round shape fruits and has been used as the genetic background of the mutagenesis experiments. LA1589 accession of *S. pimpinellifolium* (https://tgrc.ucdavis.edu/Data/Acc/AccDetail.aspx?AccessionNum=LA1589&contains=false accessed on 1 August 2022) produces small, round, red fruits and was used as a pollen donor to obtain F_2_ interspecific populations derived from its cross with the mutant line UAL-7334 for mapping purposes. Seeds of the mutants *lutescent1* (*l1*) and *lutescent2* (*l2*) were kindly provided by Cornelius Barry (Michigan State University, East Lansing, MI, USA). The phenotypic characterization of M_1_ and M_2_ plants was performed along with control MM plants under greenhouse conditions and trough cultivation cycles between spring 2014 and autumn 2018, following standard management practices, including regular fertilization. All materials generated during the current study are available from the corresponding author under a material transfer agreement.

### 4.2. EMS Mutagenesis

Mutant collection development began with M_0_ seeds mutagenesis. Initially, the mutagen median lethal dose (LD_50_) was established as a preliminary step. Briefly, seeds were incubated in 100 mL of water (control) or water containing EMS (Sigma Aldrich, Merck Group, Darmstadt, Germany). Three different EMS concentration were analyzed, i.e., 0.5%, 0.7%, and 1.0% (*v*/*v*), by using five replicates of 100 seeds for each treatment. Incubation proceeded for 16 hours with a gentle shake at 30 °C, and afterward, seeds were extensively washed in distilled water and sown for M_1_ plant germination. The M_1_ plants were cultivated under greenhouse conditions, and once they reached the fruit ripening phase, seeds from at least 10 fruits of every single M_1_ plant were collected. The collected M_2_ seeds were incubated for 12 h in a solution containing 1% of hydrochloric acid (*v*/*v*) and then washed with distilled water for 10 min. The seeds were completely dried at room temperature and then incubated for 24 h in an oven at 80 °C for virus elimination. The effect of the EMS treatments was calculated as the percentage of the seeds that died or that germinated but produced sterile plants. After the LD_50_ was calculated, a large-scale mutagenesis experiment was performed at 0.7% of EMS concentration in 16,104 MM seeds. 

### 4.3. Phenotypic Characterization

Mutants detected during the EMS collection screening were grouped into fourteen main categories, including plant vegetative and reproductive development traits which are depicted in Table 1. Statistical analysis of the inheritance pattern of each mutant phenotype detected was assessed by means of a Chi-square test, and those lines that did not fit a Mendelian inheritance pattern were considered to have complex inheritance. Regarding mutant lines that developed unfertile flowers, pollen viability assays were carried out by in vitro staining of pollen grains in a 0.5% solution of 2,3,5-triphenil tetrazolium chloride (TTC) prepared in a 0.5 M solution of sucrose, followed by two hours of incubation at 50 ºC in darkness in a humid box. An OPTIPHOT-2 (Nikon UK Ltd., Kingston upon Thames, Surrey, UK) optical microscopy was used for results visualization. Finally, progeny tests were performed in order to determine the inheritance pattern of the mutations detected.

On the other hand, with the aim to complete phenotypic characterization of *sin* mutant cell morphology and sepal ultrastructure, Scanning Electron Microscopy was performed as previously described by Lozano et al. (1998) [60]. Briefly, flowers at the anthesis day stage from WT and mutant plants were collected and fixed in a FAEG solution (3.7% formaldehyde, 5% acetic acid, 50% absolute ethanol, and 0.5% glutaraldehyde) and stored in 70% ethanol after 72 h of incubation. Before SEM analysis, samples were dehydrated in increasing ethanol concentrations and completely dried in a CO_2_ critical point with a Bal-Tec CPD 030 CO_2_ critical point dryer (Leica Biosystems, Wetzlar, Germany). Samples were then gold coated in a Bal-Tec SCD005 sputter coater (Leica Biosystems, Wetzlar, Germany) and visualized in a Hitachi S-3500N (Hitachi High Technologies Corporation, Berkshire, UK) scanning electron microscope.

### 4.4. Mapping-by-Sequencing of Sin Mutation

Cloning and identification of the *sin* mutation were performed as described by Yuste-Lisbona et al. (2021) [34]. Briefly, an F_2_ population derived from the cross of the mutant plant with the wild relative *S. pimpinellifolium* accession LA1589 as pollen donor was obtained. Following F_2_ phenotypic characterization, plant material from mutant and WT plants was collected for DNA isolation. Genomic DNA was isolated with DNAzol^®^ Reagent Kit (Fisher Scientific S.L., Madrid, Spain) following the manufacturer´s instructions, and equimolar amounts of DNA from WT and mutant F_2_ plants were used to form two DNA pools. Illumina TruSeq protocol was followed for library generation, and sequencing was carried out in an Illumina HiSeq2000 platform (Illumina, Inc., San Diego, CA, USA). The obtained sequences have been deposited in the Sequence Read Archive database at the National Center for Biotechnology Information under BioProject accession number PRJNA868127. Alignment of paired-end reads was performed against the tomato genome reference sequence version 2.5 (ITAG2.4, Boyce Thompson Institute for Plant Research, Ithaca, NY, USA) using Bowtie2 v2.4.5 (Johns Hopkins University, Baltimore, ML, USA) [61]. Duplicated reads were removed using Picard v1.65 (Broad Institute, Cambridge, MA, USA), and for indel realignment, GATK v2.2-8 (Broad Institute, Cambridge, MA, USA) was employed [62]. VCFtools v0.1.15 (Wellcome Sanger Institute, Hinxton, Cambridgeshire, UK) was used for variant calling [63], and SAMtools v1.2 (Wellcome Sanger Institute, Hinxton, Cambridgeshire, UK) for obtaining reference and non-reference allele counts for each position of the genome. Alignment and processing statistics for sequencing read data are included in Supplementary Table S1. Finally, to determine the chromosomic region where the mutations are located, the allele frequency ratio for biallelic variants was calculated as non-reference allele counts/total allele counts using a custom script in the R environment for statistical computing (v4.0.4, The R Foundation, Vienna, Austria) [64]. The functional impact of candidate causal mutations was assessed by ANNOVAR v2019Oct24 (Center for Applied Genomics, Philadelphia, PL, USA) [65], and a custom R script was used for processing the output files. R scripts are available in a GitHub repository (https://github.com/AGR-176/Mapping-by-sequencing-in-tomato/ created 13 February 2020).

### 4.5. Co-Segregation Test

With the aim of confirming the causal relationship between the mutation identified by mapping-by-sequencing and the *sin* mutant phenotype, a co-segregation test was carried out by using a CAPS codominant marker designed to flank the mutation. In short, a PCR fragment was amplified using the left and right primers sin_genot_F (5′-TCTTGAATGCTGCCTTTCTG-3′) and sin_genot_R (5′-GCCCTCAAGATATAAAAATGCAA-3′) and then digested using the restriction enzyme *Aju*I (New England Biolabs, Ipswich, MA, USA), which do not digest the WT amplicon but produce two fragments in the mutant amplicon. PCR and digestion results were visualized in a 1% agarose gel using standard DNA markers [4].

## Figures and Tables

**Table 1:** Phenotypic classes established after the screening of the 7379 M_2_ progenies of 0.7% EMS-treated seeds.

Phenotypic Class	Category	Dominants	Recessives	Complex Inheritance ^a^	Total	Frequency (%)
I	Seedling lethality and albinism	0	243	10	253	9.04
II	Root development	5	177	13	195	6.96
III	Plant size, architecture, and branching	74	1038	84	1196	42.71
IV	Leaf morphology and color	45	21	9	75	2.68
V	Shoot apical and leaf senescence	15	17	3	35	1.25
VI	Flowering time	0	2	2	4	0.14
VII	Inflorescence architecture	11	139	33	183	6.54
VIII	Flower morphology and color	17	182	21	220	7.86
IX	Flower abscission zone	2	3	0	5	0.18
X	Fruit setting	20	35	12	67	2.39
XI	Fruit morphology/color	14	197	18	229	8.18
XII	Parthenocarpy (seedless fruits)	0	218	21	239	8.54
XIII	Fruit ripening	11	55	12	78	2.79
XIV	Cuticle/cracked fruit	8	10	3	21	0.75
	TOTAL	222	2337	241	2800	100

^a^ Developmental traits whose phenotype segregation did not fit Mendelian inheritance.

## Data Availability

The obtained sequences from the mutant and wild-type pools were deposited on the Sequence Read Archive (https://www.ncbi.nlm.nih.gov/sra/) under BioProject accession number PRJNA868127.

## Supplementary Materials

The following supporting information can be downloaded at: https://www.mdpi.com/article/10.3390/plants11192453/s1, Figure S1: Complementation test performed on several tomato mutants of the EMS collection; Figure S2: Alignment performed between wild-type (WT) and *sepal indehiscent* (*sin*) mutant proteins. Table S1. Alignment and processing statistics for sequencing read data against the tomato genome reference sequence version 2.5 (ITAG2.4).
