# Supplementary material for: A Tomato EMS-Mutagenized Population Provides New Valuable Resources for Gene Discovery and Breeding of Developmental Traits
Source: Plants (Basel). 2022 Sep 20;11(19):2453. doi: 10.3390/plants11192453 (PMC9571841; doi:10.3390/plants11192453)
Supplement: Supplementary file 1 [file plants-11-02453-s001.zip › Fonseca et al. - Tomato EMS mutant collection - Supplementary Figure S1.pdf]

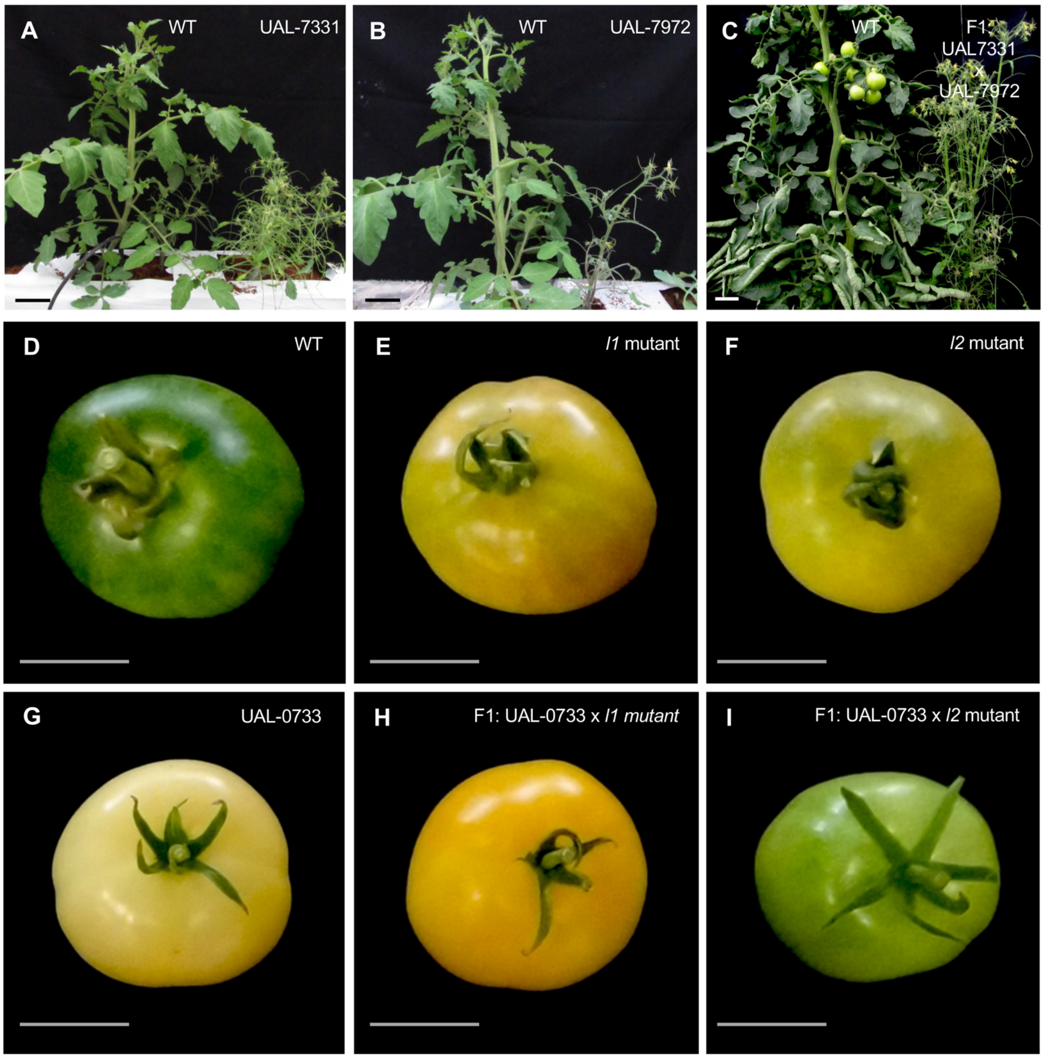

Supplementary Figure S1. Complementation test performed on several tomato mutants of the EMS collection. UAL-7331 mutant line (**A**) shows a *wiry*-like phenotype which is quite similar to that of UAL-7972 mutant line (**B**). Complementation test proved that these two lines are allelic (**C**). Fruits at mature green stage of wild-type (WT) plants (cv. Moneymaker background) (**D**); *lutescent1* (*l1*) mutant (**E**); *lutescent2* (*l2*) mutant line (**F**) and UAL-0733 mutant line (**G**). Mutant phenotype of F1 plants derived from the cross of UAL-0733 and *l1* mutants proved that both mutations are allelic (**H**). Wild-type phenotype of F_1 plants from the cross of UAL-0733 x *l2* mutant indicates genetic complementation of both mutations, demonstrating that they are located in independent loci (**I**). Scale bars: 2.5 cm.
