# Supplementary material for: A Tomato EMS-Mutagenized Population Provides New Valuable Resources for Gene Discovery and Breeding of Developmental Traits
Source: Plants (Basel). 2022 Sep 20;11(19):2453. doi: 10.3390/plants11192453 (PMC9571841; doi:10.3390/plants11192453)
Supplement: Supplementary file 1 [file plants-11-02453-s001.zip › Fonseca et al. - Tomato EMS mutant collection - Supplementary Figure S2.pdf]

```
WT      MKEVLEKCVDSQLWHACAGGMVQIPPVNSKVYYFPQGHAEHTLMNVDFSALPRSPALILC 60
sin     MKEVLEKCVDSQLWHACAGGMVQIPPVNSKVYYFPQGHAEHTLMNVDFSALPRSPALILC 60
        ************************************************************

WT      RVAAVKFLADPETDEVYAKIRVVPVGNKGNDFDDDDDILGSNESGTAEKPNSFAKTLTQS 120
sin     RVAAVKFLADPETDEVYAKIRVVPVGNKGNDFDDDDDILGSNESGTAEKPNSFAKTLTQS 120
        ************************************************************

WT      DANNGGGFSVPRYCAETIFPRLDYTADPPVQTVTAKDVHGESWKFRHIYRGTPRRHLLTT 180
sin     DANNGGGFSVPRYCAETIFPRLDYTADPPVQTVTAKDVHGESWKFRHIYRGTPRRHLLTT 180
        ************************************************************

WT      GWSSFVNQKKLVAGDSIVFLRAENGELCVGIRRAKRGGIGGPEAPSGWNSGAGNYGGFSA 240
sin     GWSSFVNQKKLVAGDSIVFLRAENGELCVGIRRAKRGGIGGPEAPSGWNSGAGNYGGFSA 240
        ************************************************************

WT      FLREEMSKNGNLTSPTRSLRGKGRVRPESVVEAAYLASSGQPFEVVYYPRANTPEFCVRA 300
sin     FLREEMSKNGNLTSPTRSLRGKGRVRPESVVEAAYLASSGQPFEVVYYPRANTPEFCVRA 300
        ************************************************************

WT      SSVNAAMRIQWCSGMRFKMAFETEDSSRISWFMGTISSIQLADPIRWPNSPWRLLQVAWD 360
sin     SSVNAAMRIQWCSGMRFKMAFETEDSSRISWFMGTISSIQLADPIRWPNSPWRLLQVAWD 360
        ************************************************************

WT      EPDLLQNVKHVSPWLVELVSNMPVIHLSPFSPPRKKLRLPPDFSLDSQFQLPSFSGNPLR 420
sin     EPDLLQNVKHVSPWLVELVSNMPVIHLSPFSPPRKKLRLPPDFSLDSQFQLPSFSGNPLR 420
        ************************************************************

WT      SSSPFCCLSDNITAGIQGARHAQFGVPLLDLHLSNKLPSGLLPPSFQRVAANSQ-----L 475
sin     SSSPFCCLSDNITAGIQGARHANLEYLYWIFTLATNYRQD----CYHQVSSALQPTHNFL 476
        **********************::      : *:.:  ..    .:::*::  *     *

WT      PNVINKCQNDRNDNISCLLTMGTSSKTLDKNDSVNTPRFLLFGQPILTEQQISNGCSVSA 535
sin     MSSISAKMTEMITSLVCLQWVLQVR---------------HWTKMIV------------- 508
         . *.   .:   .: **  :                    : : *:

WT      PQVVQTGKDLGRIQPINEKHPSEQKGSIQDNLSSATFFWNRGYHAAELGVLNTGHCKVFL 595
sin     ------------------------------------------------------------ 508

WT      ESEDVGRTLDLSVMGSYEELYKRLANMFGLERPDMLTRVLYHDATGAVKHTGDEPFSDFV 655
sin     ------------------------------------------------------------ 508

WT      KSAKRLTILMNSSSNIKRKWLTGLATAERGLDSSNQAGPLSIFA                 699
sin     --------------------------------------------                 508
```

Supplementary Figure S2. Alignment performed between wild-type (WT) and sepal indehiscent (sin) mutant proteins. The DNA B3 binding domain and the Auxin Response Factor domain (middle region) appear underlined by a solid and dashed line respectively. Sl-miRNA160a target sequence is highlighted in gray. Nuclear Localization Signal (NLS) motifs appear in bold. Finally, type III Aux/IAA domain appears in red whereas type IV Aux/IAA is displayed in bold red letters.
