# Supplementary material for: A Tomato EMS-Mutagenized Population Provides New Valuable Resources for Gene Discovery and Breeding of Developmental Traits
Source: Plants (Basel). 2022 Sep 20;11(19):2453. doi: 10.3390/plants11192453 (PMC9571841; doi:10.3390/plants11192453)
Supplement: Supplementary file 1 [file plants-11-02453-s001.zip › Fonseca et al. - Tomato EMS mutant collection - Supplementary Table S1.pdf]

Supplementary Table S1. Alignment and processing statistics for sequencing read data against the tomato genome reference sequence version 2.5 (ITAG2.4).

	**Moneymaker**[a]	***sin* wild-type pool**[b]	***sin* mutant pool**[c]
Total sequencing reads	63,575,958	94,007,596	99,685,109
Uniquely mapped reads	56,571,716 (88.98 %)	88,279,910 (93.91 %)	92,975,595 (93.27 %)
Multi-mapped reads	1,032,320 (1.62 %)	1,355,151 (1.44 %)	1,496,688 (1.50 %)
Unmapped reads	5,971,922 (9.39 %)	4,372,535 (4.65 %)	5,212,826 (5.23 %)
Sequencing depth	15.40 X	34.02 X	36.04 X
Sequence variants	813,450	6,100,013	6,707,616

[a] NCBI SRA BioSample accession: SAMN04093174.

[b] NCBI SRA BioSample accession: SAMN30553549.

[c] NCBI SRA BioSample accession: SAMN30553548.
